# Comparison of n-3 PUFA-Enriched vs. Olive-Oil-Based Lipid Emulsion on Oxidative Stress and Inflammatory Response in Critically Ill Post-Surgery Adults: Secondary Analysis of a Randomized Controlled Trial

**DOI:** 10.3390/ijms252111739

**Published:** 2024-10-31

**Authors:** Nerea Cuartero-Corbalán, Fátima Martínez-Lozano Aranaga, Maria Jesús Gómez-Ramos, María B. Gómez-Sánchez, Francisco V. Avilés-Plaza, María A. Núñez-Sánchez, Juana M. Morillas-Ruiz

**Affiliations:** 1 Faculty of Pharmacy and Nutrition, Campus de Los Jerónimos, Universidad Católica San Antonio de Murcia, Guadalupe, 30107 Murcia, Spain; ncuartero@alu.ucam.edu (N.C.-C.); jmmorillas@ucam.edu (J.M.M.-R.); 2 Intensive Care Unit, Hospital General Universitario Reina Sofía, Avda Intendente Jorge Palacios 1, 30003 Murcia, Spain; 3 Nutrition Unit, Hospital General Universitario Reina Sofía, Avda Intendente Jorge Palacios 1, 30003 Murcia, Spain; mbienvenida.gomez@carm.es; 4 Department of Clinical Analysis, Hospital General Universitario Virgen de la Arrixaca, 30120 Murcia, Spain; franciscov.aviles@carm.es; 5 Obesity, Diabetes and Metabolism Laboratory, Biomedical Research Institute of Murcia-Pascual Parrilla (IMIB-PP), Crt. Buenavista s/n, 30120 Murcia, Spain

**Keywords:** parenteral nutrition, intravenous lipid emulsion, inflammation, oxidative stress, critically ill patients

## Abstract

Malnutrition in critically ill patients represents a major concern as it can lead to adverse outcomes including increased morbidity and mortality. These patients exhibit an impaired immune response accompanied by increased oxidative stress. Nutritional support, including parenteral nutrition (PN), is critical in these patients. Intravenous lipid emulsions (ILEs), a key component of PN, provide energy and intervene in the modulation of inflammation. This was a secondary study of a randomized clinical trial at the Reina Sofia University Hospital (Murcia, Spain) for critically ill patients following major abdominal surgery that were administered PN supplemented with olive-oil-based ILE (OO-ILE, *n* = 29) or a mixed-lipid ILE (soybean oil, medium chain triglycerides, OO and fish oil, SMOF-ILE, *n* = 25). The effects on clinical outcomes, metabolic markers, oxidative stress, and inflammation were evaluated. No significant differences were observed between groups in the clinical parameters and outcomes, oxidative stress, or inflammatory markers. The within-group evaluation demonstrated an increase in total antioxidant capacity in both groups, while OO-ILE increased the levels of 15-F2t-isoprostane. In addition, the results showed that both mixtures reduced the release of IL-1β and IL-6. These findings suggest that both treatments had similar effects on oxidative stress and inflammatory response in this type of patient.

## 1. Introduction

Malnutrition in hospitalized patients is a major concern for health systems. It is estimated that it affects between 20% and 50% of total hospitalized patients [1] and it could affect up to 78% of intensive care unit (ICU) patients [2]. Specifically, critically ill patients following major abdominal surgery are known to be at a high risk of malnutrition, which is associated with several adverse outcomes such as a higher rate of infections, longer hospital and ICU stays, and increased morbidity and mortality [3]. These patients are known to have alterations in immune response, including an exacerbated release of inflammatory cytokines, which in turn could lead to cellular damage, hypoxia, hypoperfusion, and endothelial damage, which is accompanied by increased oxidative stress due to the presence of a large amount of free radicals [4]. All this is characterized by an enhanced breakdown of endogenous proteins and an intolerance to carbohydrates, together with a hypermetabolic state. Therefore, it is critical to provide nutritional support to these patients, either through enteral or parenteral nutrition (PN), based on the clinical status of the patient [5]. In this regard, PN is usually indicated for critically ill patients after abdominal surgery due to the hypercatabolic state combined with the necessity for extended periods of intestinal rest, which is frequently required [6].

Lipids are a fundamental component of PN, not only providing both an energy-dense source of calories and essential fatty acids but also playing a structural role in cell signaling, participating in several enzymatic functions, and as precursors for several pro- and anti-inflammatory lipid mediators [7]. Intravenous lipid emulsions (ILEs) have been used in PN since 1961, which initially were based on lipids from pure soybean oil. However, these ILEs have been suggested to be associated with a number of adverse effects such as increased inflammatory response and suppressed cell-mediated immunity [8,9,10]. These detrimental effects have been related to their high content in n-6 polyunsaturated fatty acids (PUFAs), especially in linoleic acid, which is transformed into arachidonic acid, a precursor of prostaglandin, leukotriene, and thromboxane [11].

Over the years, different formulations, such as mixtures of soybean oil and medium-chain triglycerides (MCT), olive oil (OO), and/or fish oil (FO), have been explored in order to mitigate the inflammatory response as well as to decrease oxidative stress, thus improving the clinical outcomes in patients requiring PN [12,13,14,15,16]. In this regard, a number of studies have shown that the use of these ILEs in PN has beneficial effects compared to traditional soybean-based ILEs [17]. For instance, OO-based ILEs, which are rich in monounsaturated fatty acids (MUFAs), primarily oleic acid, have been described to reduce ICU length of stay, days of mechanical ventilation, and infection compared to traditional ILEs [18,19]. Similarly, FO-containing ILEs, which represent a lipid source rich in n-3 PUFAs such as eicosapentaenoic acid and docosahexanoic acid, have also been demonstrated to reduce the incidence of infections and overall morbidity and mortality [20]. However, while most of the studies have focused on critically ill patients independently of the admission cause to ICU, the effects on the clinical outcomes, oxidative stress, and inflammation of patients admitted to ICU after abdominal surgery are still scarce [21]. Furthermore, these studies have focused on the differences between traditional SO-ILEs and those containing different mixtures of lipids, and less is known about the differences between OO-ILEs and FO-containing ILEs [20,22,23].

Thus, in the present study, we aimed to evaluate the effect of two different types of ILEs, one based on OO (OO-ILE) and the other containing a mixture of soybean oil, MCT, OO, and FO (SMOF-ILE), on oxidative stress markers and inflammatory response. Furthermore, we also investigated whether the composition of lipids in PN affected the course of clinical outcomes such as infections, hospital and ICU length of stay, mechanical ventilation, and mortality, as well as on hepatic and lipid metabolism markers.

## 2. Results

### 2.1. Characteristics of the Study Population

There were no significant differences between the baseline anthropometric characteristics of the two groups (Table 1). Similarly, the severity scores measured by the APACHE II tests were similar in both groups. The primary reasons for ICU admission were patients with acute abdominal pain undergoing programmed surgery and with surgical complications such as hypovolemic shock (*n* = 2), systemic inflammatory response syndrome (*n* = 41), sepsis (*n* = 6), or surgical reintervention within the first 24 h after the first surgery (*n* = 5).

### 2.2. Effects on the Clinical Parameters and Outcomes of the Patients

The levels of the different biochemical and clinical parameters evaluated showed that, except for basal levels of aspartate transaminase (AST), there were no differences between the OO-ILE and SMOF-ILE groups at baseline or seven days after the initiation of PN (Supplementary Table S1). The analysis of the within-group differences showed a statistically significant increase in the serum levels of prealbumin, transferrin, triglycerides, cholesterol, apolipoprotein AI, and apolipoprotein B, while the SOFA score significantly decreased in both groups independently of the lipid source administered in PN (Table 2). Patients who were administered OO-ILE showed an increase in albumin levels and a decrease in AST (Table 2). However, the between-groups comparison revealed that the effects observed were not dependent on the type of treatment as there were no significant differences in the evolution of the parameters analyzed between the OO-ILE and SMOF-ILE groups.

Patients were monitored on a daily basis for the days of need for mechanical ventilation, days with a central venous catheter, ICU and hospital length of stay, number of days of PN, and complications such as ventilator-associated pneumonia or exitus, as shown in Table 3. No significant differences were observed in the evolution of the clinical outcomes between the two groups.

### 2.3. Effects of Lipid Composition on Serum Oxidative Stress Markers

**Figure 1:**
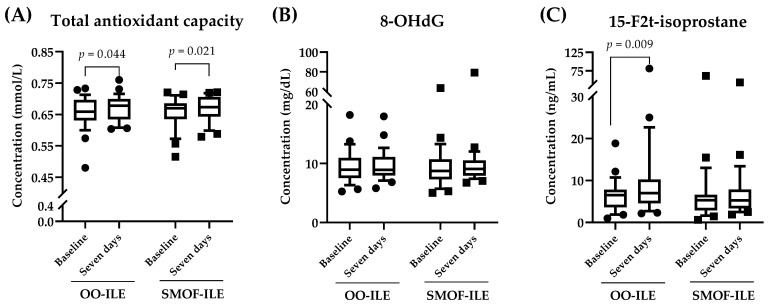
Serum levels of oxidative stress markers. The results are represented as box plot graphs showing the distribution of (**A**) total antioxidant capacity; (**B**) 8-hydroxyguanosine (8-OHdG); and (**C**) 15-F2t-isoprostane levels at the different time points. The central line in each box represents the median, while the edges of the box denote the interquartile range (IQR). All variables were assessed for normality using a Shapiro–Wilk normality test. Within–between interactions (treatment × time) were analyzed by a mixed-model ANOVA, and within-group variations were calculated using a Wilcoxon signed-rank test (considering *p* < 0.05 significant). OO-ILE, olive-oil-based intravenous lipid emulsion; SMOF-ILE, soybean oil, medium chain triglycerides, olive oil, and fish oil intravenous lipid emulsion.

To determine whether the treatments had an effect on the biological redox state of the patients, we next evaluated the oxidative stress response to both treatments. The analysis showed no statistically significant differences between the two groups at baseline or seven days after the treatment regarding total antioxidant capacity (measured by the Trolox equivalent antioxidant capacity test, TEAC), as well as in the levels of 8-hydroxyguanosine (8-OHdG) and 15-F2t-isoprostane (Figure 1). The within-group analysis showed that while none of the treatments had an effect on 8-OHdG levels, there was a significant increase in total antioxidant capacity after seven days independent of the treatment. On the other hand, the analysis of 15-F2t-isoprostane levels indicated that while the treatment with SMOF-ILE had no significant effect after 7 days (5.30 (3.77) ng/mL at baseline vs. 5.24 (4.54) ng/mL after seven days, *p* = 0.065), the levels of 15-F2t-isoprostane significantly increased in the OO-ILE group (6.5 (4.21) ng/mL vs. 6.99 (5.62) ng/mL).

### 2.4. Effects of Lipid Composition on Serum Inflammatory Markers

**Figure 2:**
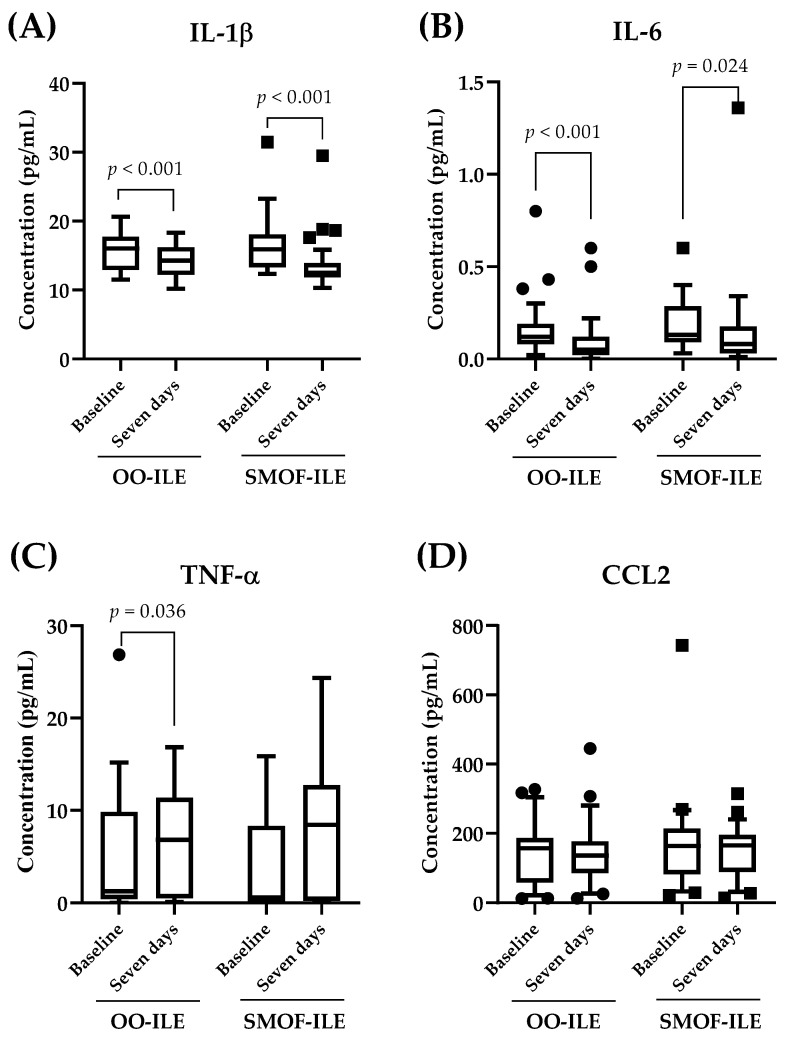
Serum levels of inflammatory markers. The results are represented as box plot graphs showing the distribution of (**A**) IL-1β; (**B**) IL-6; (**C**) TNF-α; and (**D**) CCL2 levels at the different time points. The central line in each box represents the median, while the edges of the box denote the interquartile range (IQR). All variables were assessed for normality using a Shapiro–Wilk normality test. Within–between interactions (treatment × time) were analyzed by a mixed-model ANOVA, and within-group variations were calculated using a Wilcoxon signed-rank test (considering *p* < 0.05 significant). CCL2; C-C motif ligand 2; IL-1β, interleukin 1 beta; IL-6, interleukin 6; OO-ILE, olive-oil-based intravenous lipid emulsion; SMOF-ILE, soybean oil, medium chain triglycerides, olive oil, and fish oil intravenous lipid emulsion; TNF-α, tumor necrosis factor alpha.

The analysis of inflammatory markers showed that there were no significant differences in serum cytokines between the groups at any of the time points studied (Figure 2A–D). Similarly, the levels of C-C motif chemokine 2 (CCL2) remained similar after seven days of PN independent of the lipid composition in the PN (Figure 2D). On the other hand, the intragroup analysis showed that both treatments were equally effective in reducing the levels of interleukin 1β (IL-1β) and interleukin 6 (IL-6) after seven days (Figure 1A,B). Interestingly, while there were no differences between tumor necrosis factor α (TNF-α) levels before and after PN in the group treated with SMOF-ILE, there was a statistically significant increase in TNF-α after seven days of treatment with OO-ILE (Figure 1C, 1.26 (9.42) pg/mL at baseline vs. 6.82 (10.87) pg/mL after seven days of treatment).

## 3. Discussion

In the present study, we have evaluated the differences between two different ILEs in PN on the clinical and biochemical parameters of adult critically ill patients after major abdominal surgery, as well as their effect on oxidative stress and inflammatory markers. Our results showed that there were no differences observed in the clinical and biochemical parameters analyzed between the two treatments. In addition, the analysis of oxidative stress markers showed that both treatments were effective in increasing total antioxidant capacity, while no changes were observed in 8-OHdG. On the other hand, the levels of 15-F2t-isoprostane were significantly increased in patients of the OO-ILE group. The evaluation of anti-inflammatory markers showed that both treatments significantly reduced the levels of IL-1β and IL-6. Interestingly, patients exhibited an increase in TNF-α levels after the treatment, although such an increase was only significant among those patients that were administered OO-ILE.

Over the past several years, expert associations such as the European Society for Clinical Nutrition and Metabolism (ESPEN) or the American Society for Parenteral and Enteral Nutrition (ASPEN) have been encouraging the use of n-6 PUFA-reduced ILEs as alternatives to traditional soybean-oil ILEs [24,25,26]. These recommendations arise from the available evidence indicating that n-6 PUFAs are able to increase lipid peroxidation, resulting in an exacerbated oxidative stress response and impaired inflammatory response, which in turn could translate into a worsened clinical course and outcome [17]. Thus, the use of alternative lipid sources, such as OO or FO, has been demonstrated to decrease infections and shorten hospital and ICU lengths of stay, with a trend of reduced 28-day mortality [19,27,28]. However, although n-3 PUFA-enriched ILEs are the preferred source of lipids in PN, the superiority of these ILEs over OO-based ILEs in terms of clinical course and outcomes in adult critically ill patients has yet to be determined. In this study, we have shown that there were no significant differences between the two treatments after seven days in any of the parameters included. These results are consistent with previous results reported in a limited number of clinical trials. Thus, studies comparing the efficacy of OO-ILEs vs. FO-containing ILEs in adult patients have shown that mortality, hospital and ICU length of stay, or SOFA score remained similar independent of the lipid source employed, highlighting that higher concentrations of n-6 PUFAs may contribute to a poorer clinical course in these patients [20,22,29]. Of note, in the study performed by Badía-Tahull et al. [22], the authors reported a significantly higher incidence of infections in post-surgical patients that were administered OO-ILE in comparison with those receiving SMOF-ILE (78% vs. 23%, respectively). Nonetheless, this study was carried out in post-surgical patients with or without the need of ICU admission and with a small sample size (14 patients with OO-ILE vs. 13 patients with SMOF-ILE) [22]. Similarly, studies of patients on home PN or post-surgical patients have indicated that SMOF-ILEs may have a beneficial effect on liver function compared to OO-ILEs by reducing AST, alanine aminotransferase (ALT), and α-glutathione S-transferase levels [30,31]. In our study, not only did we not find differences between the treatments, but we observed a modest yet statistically significant reduction in AST levels in those patients receiving OO-ILE. These discrepancies could be due to different factors, such as the heterogeneity of the type of patient, as well as the differences in the treatments and timelines that may reduce the applicability of these findings, which makes it challenging to establish clear differences in the clinical outcomes and clinical course between OO-ILE and SMOF-ILE in our targeted population.

Critically ill patients after major abdominal surgery are particularly vulnerable to oxidative stress and inflammation due to the interplay of several factors [5]. Surgical trauma initiates an inflammatory response and increases the production of reactive oxygen species (ROS) together with a reduction in the ability of the endogenous antioxidant defense system to neutralize them. Elevated levels of these compounds have been correlated with poor clinical outcomes in adult critically ill patients, such as sepsis, multiple organ dysfunction syndrome, and mortality [32]. Thus, interventions aimed to reduce oxidative stress such as nutritional strategies have arisen as promising therapies to improve the antioxidant response in critically ill patients [16]. In this regard, in recent years, it has been suggested that the use of ILEs, especially those enriched in n-3 PUFAs, could potentially reduce the production of ROS [13]. Here, we have compared the effects of two different ILEs on oxidative stress by analyzing three different markers: total antioxidant capacity, 8-OHdG, and 15-F2t-isoprostane. Our results showed that there were no differences between the markers studied independently of the ILE used. Interestingly, when analyzing the within-group differences, we observed that while both ILEs were associated with a positive effect on total antioxidant capacity, those supplemented with OO-ILE exhibited an increase in the levels of circulating 15-F2t-isoprostane after seven days, which remained unchanged in the SMOF-ILE group. The observed increase in 15-F2t-isoprostane in the OO-ILE suggests a more protective effect of SMOF-ILE against oxidative stress in this type of patient. This could be potentially due to differences in the fatty acid composition or antioxidant content between both ILEs [33]. These results differ from those reported by other authors where the use of OO-ILEs was described to decrease the levels of antioxidant markers compared to other types of ILEs [34]. However, these studies were performed using different antioxidant markers (i.e., oxidized LDL, TBARS, and TAS) and in a different population of patients. On the other hand, other authors have described that SMOF-ILE supplementation significantly reduces malondialdehyde levels on home PN patients [12]. All this suggests that the impact of the different ILE formulations on oxidative stress may vary based on the specific underlying conditions and metabolic status of the population studied, highlighting the necessity for further studies.

Finally, we also evaluated the differences between the two types of ILEs regarding inflammatory response. Over the past years, soybean-oil-based ILEs have been shown to promote the production of pro-inflammatory cytokines, probably due to the high content in linoleic acid, which in turn acts as a precursor of arachidonic acid, leading to the production of eicosanoids which might predispose a more pro-inflammatory response [16]. Thus, the use of alternatives with reduced n-6 PUFA content such as OO-ILE or SMOF-ILE has been preferred in clinical recommendations [26,35,36]. Here, we have shown that both OO-ILE and SMOF-ILE had a comparable effect on the inflammatory markers studied, with no differences between the groups after seven days on PN. Furthermore, the intragroup analysis showed that the levels of IL-1β and IL-6 underwent a modest yet significant reduction in both groups after seven days. In this regard, the observed reduction in these markers in those patients treated with SMOF-ILE is consistent with previous observations where the enrichment in n-3 PUFAs has been related to a better evolution of inflammatory status in adult septic critically ill patients [29]. This reduction in inflammatory markers could be due to a balanced lipid profile and the contents in eicosapentaenoic acid and docosahexaenoic acid, which are known to downregulate pro-inflammatory pathways via competing with arachidonic acid [37]. On the other hand, the effect of OO-ILE administration on the inflammatory response of adult patients is less clear. A previous study performed in adults on home PN showed that OO-ILE had a better effect on some markers of inflammation when compared to SMOF-ILE [12]. Contrastingly, other studies performed in critically ill patients showed that OO-ILE had similar effects on cytokine release compared to soybean-based ILE [38,39]. Here, we showed that both treatments exerted similar effects on the modulation of the inflammatory response, although further studies are needed to confirm such observations in this type of patient.

We acknowledge that our study has some limitations but also some strengths. One limitation is the small number of participants included in this study as it was performed in a single hospital and the inherent nature of the patients included in the study (adult critically ill patients after major abdominal surgery). In addition, some of the participants enrolled did not reach the goal of seven days on PN, either because it was no longer required or due to mortality. Another potential limitation is the number of days selected, seven days. This limitation might be overcome by extending the time of the study, which is challenging as only a very limited number of patients remained in ICU on PN after this period of time. On the other hand, our study also has some strengths. For instance, the distribution of groups was well-balanced, and the baseline parameters were similar between the groups. In addition, the inclusion of a large number of parameters provided a more comprehensive understanding of the potential effects of the different types of ILEs. Also, we did not observe significant differences between any of the parameters analyzed at baseline after randomization. All this minimized potential bias in the study and allowed us to reliably assess for differences in the progression of such parameters after treatment.

All in all, further research is essential to confirm the findings of this study and clarify the specific effects of OO-ILE and SMOF-ILE on oxidative stress and inflammation in critically ill patients after major abdominal surgery. Given that our study did not reveal significant differences between the two ILEs, future randomized controlled trials with larger numbers of patients are necessary to confirm these results. In addition, increasing the duration of treatment could also help to better evaluate the long-term effects of these ILEs on clinical outcomes. Also, exploring the potential mechanisms behind the observed increase in isoprostanes with OO-ILE will be crucial to better understand the benefits and risks of each formulation. Increasing our understanding of the specific effects of OO-ILE and SMOF-ILE could contribute to improved guidelines for ILE administration in critically ill patients and therefore enhance overall patient care.

## 4. Materials and Methods

### 4.1. Study Design

This study is a secondary analysis of data from a prospective, single-blind, two-arm, parallel, randomized, active controlled, and non-inferiority phase 3 clinical trial that examined the effectiveness and safety of two ILEs for PN in post-surgical critically ill patients [23]. In the main study, participants were randomized to receive either OO-ILE or SMOF-ILE in PN. Assessment took place at baseline, every week, at ICU discharge, at hospital discharge, and one and six months after hospital discharge [23]. The primary outcomes of the main study were to determine the effectiveness in clinical outcomes determined by the reduction in nosocomial infections and the safety determined by the overall mortality. Secondary outcomes included ICU and hospital length of stay, days on MV, and determination of hypertriglyceridemia and hepatic enzymes [23]. The Consolidated Standards of Reporting Trials (CONSORT) checklist can be found in Supplementary Table S1.

### 4.2. Sample Size, Recruitment, and Participants

**Figure 3:**
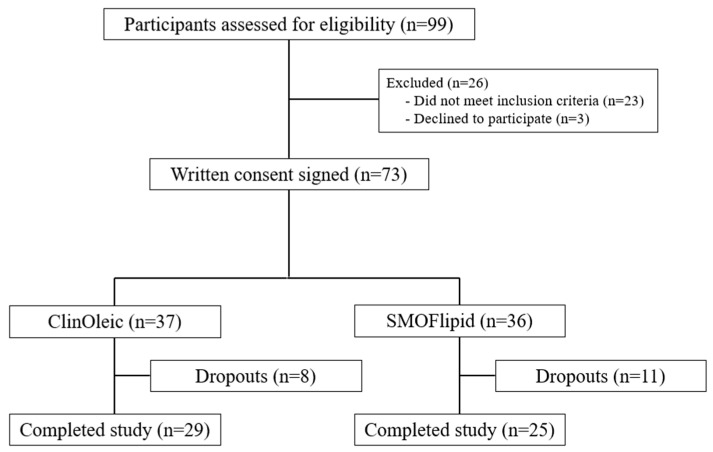
Flow diagram of patients included in the study (CONSORT diagram).

A total of 99 adult critically ill post-surgical patients were assessed for eligibility at the ICU of the Reina Sofia University Hospital of Murcia (Murcia, Spain) between May 2014 and December 2017. Written informed consent was obtained from the patients (or their relatives for those unable to provide consent due to the severity of their condition). The inclusion criteria included age > 18 years and post-surgical patients who were admitted to the ICU that required PN for at least seven days. The exclusion criteria included patients with an expected stay in the ICU of less than 7 days, patients with morbid obesity (BMI ≥ 40 kg/m^2^), chronic renal failure (creatinine > 7 mg/dL or chronic dialysis), hyperlipidemia, autoimmune disease, severe liver disease (cirrhosis or Child–Pugh score > 3), severe acute pancreatitis, occurrence of neoplasia with metastasis, patients who had undergone chemotherapy or radiotherapy within one month prior to ICU admission, or subjects who declined to participate. The flow of participants is shown in Figure 3.

This study was carried out in agreement with the Declaration of Helsinki according to local and national laws and was approved by the Ethics and Clinical Research Committees of the Reina Sofia University Hospital of Murcia (approved in January 2014, Internal Reference 1-2014).

### 4.3. Nutritional Intervention

A total of 73 patients (or their legal representatives) gave their written consent and were randomly allocated in a 1:1 ratio to two groups based on the lipid source used in the PN. Randomization was performed using a computer-generated blocked-randomization scheme to ensure a balance in sample size between the groups. Both groups received the same daily caloric (20–30 kcal/kg/d), nitrogen (0.2–0.3 g/kg/d), and lipid (0.8 g/kg/d) intake. Mineral and vitamin composition were identical for both groups. Patients were administered PN supplemented with either OO-ILE (*n* = 37), which was composed of olive oil (OO, 80%) and refined soybean oil (20%) (ClinOleic, Baxter S.L., Valencia, Spain), or SMOF-ILE (*n* = 36) based on a lipid solution consisting of soybean oil (30%), medium-chain triglycerides (30%), OO (25%), and fish oil (FO) (15%) (SMOFlipid, Fresenius Kabi, Barcelona, Spain). Both solutions were prepared daily following the standardized protocols of the Reina Sofía University Hospital of Murcia and were administered through a central venous catheter as a 24 h infusion. All patients received PN adjusted to their body weight, following the recommendations for specialized nutritional and metabolic support for critically ill patients established by the Spanish Society of Intensive Medicine Metabolism and Nutrition group [32]. For the purpose of this secondary study, only patients with an ICU length of stay of at least 7 days were included. Thus, twenty-nine and twenty-five patients from group 1 and group 2, respectively, were included in this secondary analysis, as shown in the CONSORT diagram (Figure 3).

### 4.4. Clinical and Demographic Data Collection

All data regarding the characteristics of the patients were collected at baseline after obtaining signed written consent, including age, sex, body mass index (BMI), and reasons for the admission to ICU. Data of the indicators of severity of illness were collected using the severity scales Acute Physiology and Chronic Health Evaluation (APACHE II) and sepsis-related organ failure assessment (SOFA). The SOFA test was performed every 7 days until ICU discharge. Documentation of vital signs, infection occurrence, and monitoring of catheters (urinary and central), as well as the composition of PN, was carried out daily. Data regarding clinical outcomes such as hospital and ICU length of stay, days of mechanical ventilation, and in-hospital mortality were also obtained. Mortality rates were evaluated at hospital discharge and six months after ICU admission via medical record or telephone call.

### 4.5. Biological Samples and Laboratory Analyses

Blood and urine samples from study patients were collected on the day of ICU admission (baseline) and one week after the start of PN administration. The determination of albumin, pre-albumin, transferrin, gamma-glutamyl transferase (GGT), alanine aminotransferase (ALT), aspartate transaminase (AST), bilirubin, apolipoprotein A-I, and apolipoprotein B was performed in serum and urine samples using the Advia Chemistry XPY system (Siemens, Madrid, Spain). The complete blood count was performed in whole blood using a DxH 800 hematology analyzer (Beckman Coulter, Nyon, Switzerland).

### 4.6. Determination of Inflammatory and Oxidative Stress Biomarkers in Serum Samples

The analysis of inflammatory cytokines was performed in serum samples using enzyme-linked immunosorbent assay (ELISA) kits following the manufacturer’s instructions. The ELISA kits for pro-inflammatory cytokines CCL2 and TNF-α were obtained from Aviscera Bioscience (Santa Clara, CA, USA) and Hycultbiotech (Uden, The Netherlands), respectively. ELISA kits for IL-1β and IL-6 were acquired from Assaypro (St Charles, MO, USA). The ELISA kits for the determination of oxidative stress biomarkers 15-F2t-isoprostane and 8-OHdG were acquired from Oxford Biomedical Research (Rochester Hills, MI, USA) and JaICA (Shizuoka, Japan), respectively. The absorbance was measured using the DS2^®^ 2-Plate ELISA Processing System (Dynex™ Technologies, Chantilly, VA, USA) and analyzed using the DS-Matrix™ control software v.1.0.27. The concentration of each biomarker was obtained by using the corresponding standard curve.

### 4.7. Trolox Equivalent Antioxidant Capacity Assay

Total antioxidant capacity was evaluated using the TEAC (Trolox Equivalent Antioxidant Capacity) assay as previously described with some modifications [40]. Briefly, an ABTS+ (2,2′-azino-bis-3-ethylbenzothiazoline-6-sulfonic acid) solution containing 5 mL of 7 mM ABTS and 88 µL of 140 mM of potassium persulfate was prepared. The concentration of the ABTS radical was adjusted with methanol to an initial absorbance of 0.7 at 734 nm before measuring the samples. A total of 100 µL of serum sample was added to 1 mL ABTS+ radical solution, incubated at room temperature for 30 min, and absorbance was measured at 734 nm using a Cytation3 Multimode Reader (BioTek, Winooski, VT, USA). The standard curve was generated using Trolox as an antioxidant (0–1.5 mmol Trolox/L).

### 4.8. Statistical Analysis

Data were analyzed using SPSS 28.0.1.1 (IBM, New York, NY, USA). The normal distribution of the study variables was assessed using a Shapiro–Wilk test. Data following a normal distribution are presented as the mean ± SD, while data that failed to follow normality are presented as the median (IQR). The analysis of categorical data was carried out by a Chi-squared test for intra-group differences and a Pearson’s Chi-squared test for inter-group differences. For the quantitative data, a *t*-test was used for those parameters following a normal distribution, while a Wilcoxon signed-rank test was used for those without a normal distribution to evaluate intra-group differences. Within–between interaction (treatment × time) group differences were analyzed by a mixed-model repeated measures ANOVA with time and treatment as fixed factors. A significance of <0.05 was used throughout the analysis. Figures were represented using Prism 8.0 (GraphPad Software Inc., La Jolla, CA, USA).

## 5. Conclusions

Summarizing, our results showed that there were no differences between the administration of OO-ILE and SMOF-ILE in the clinical evolution of adult critically ill patients in terms of their biochemical parameters and clinical outcomes. Similarly, we did not find differences between the treatments in the concentration of oxidative stress and inflammatory markers. However, the within-group analysis revealed that the use of OO-ILE had a negative effect on the concentration of isoprostanes, indicating an increase in oxidative stress. In addition, both OO-ILE and SMOF-ILE demonstrated a reduction in the concentration of inflammatory markers after seven days of treatment. These findings suggest that both ILEs exert similar effects in overall outcomes, although SMOF-ILE might offer some advantages in terms of modulating oxidative stress in adult critically ill patients after major abdominal surgery. Nevertheless, more randomized controlled trials are needed to confirm these observations in order to optimize PN strategies for these types of patients.

## Figures and Tables

**Table 1:** Characteristics of the study population at baseline.

	Total (*n* = 54)	OO-ILE (*n* = 29)	SMOF-ILE (*n* = 25)	*p*
Gender (M/F)	36/18	17/12	19/6	0.249
Age (years)	68.9 ±13.5	69.2 ± 15.1	68.6 ± 11.7	0.864
Weight (kg)	75.0 (11.5)	75.0 (10.0)	75.0 (17.0)	0.379
BMI	27.7 ± 5.3	27.3 ± 5.9	28.1 ± 4.5	0.570
APACHE II	15.0 (9.5)	14.0 (8.8)	17.0 (13.5)	0.848

Values are presented as the mean ± standard deviation (SD) for those quantitative variables following a normal distribution (age and BMI) and as the median (IQR) for those without a normal distribution (weight and APACHE II). *p* values were calculated to evaluate differences between treatments using a *t*-test for independent samples in those parameters with a normal distribution, whereas *p* values for parameters without a normal distribution were calculated using a U Mann–Whitney test. Values for sex are presented as frequencies. *p* values were calculated using a Pearson’s Chi-squared test. *p* < 0.005 was considered statistically significant. APACHE II, acute physiology and chronic health disease classification system II; BMI, body mass index; IQR, interquartile range; OO-ILE, olive-oil-based intravenous lipid emulsion; SMOF-ILE, soybean oil, medium-chain triglycerides, olive oil, and fish oil intravenous lipid emulsion.

**Table 2:** Biochemical parameters before and after 7 days of PN of patients receiving OO-ILE and SMOF-ILE.

Variable	OO-ILE (*n* = 29)			SMOF-ILE (*n* = 25)			Interaction Treatment × Time
	Baseline	7 Days	*p*	Baseline	7 Days	*p*	*p*
Albumin (g/dL)	2.1 ± 0.5	2.3 ± 0.5	**0.019**	2.1 ± 0.4	2.2 ± 0.5	0.473	0.361
Prealbumin (mg/dL)	7.2 (5.9)	14.9 (9.6)	**<0.001**	7.8 (1.7)	14.3 (10.8)	**<0.001**	0.331
Transferrin (mg/dL)	97.7 ± 26.6	113.6 ± 39.1	**0.010**	99.3 ± 31.1	119.5 ± 28.9	**0.004**	0.262
Triglycerides (mg/dL)	94.0 (70.3)	170.0 (90.3)	**0.001**	104.0 (65.0)	141.0 (79.5)	**0.041**	0.505
Cholesterol (mg/dL)	83.5 (56.2)	110.0 (47.0)	**0.006**	80.0 (15.0)	101.0 (49.0)	**0.003**	0.330
GGT (U/L)	65.0 (57.0)	86.0 (91.0)	0.347	86.5 (68.0)	95.0 (79.7)	0.737	0.616
AST(U/L)	16.0 (13.0)	28.0 (38.0)	**0.046**	24.5 (42.2)	32.0 (22.4)	0.476	0.584
ALT(U/L)	22.0 (31.5)	39.0 (42.0)	0.078	22.5 (42.2)	28.0 (28.8)	0.382	0.518
Bilirubin (mg/dL)	0.33 (0.28)	0.45 (0.65)	0.256	0.52 (0.72)	0.59 (0.68)	0.830	0.400
Apolipoprotein AI (mg/dL)	58.3 ± 18.7	72.5 ± 18.2	**<0.001**	50.8 ± 20.1	65.2 ± 18.7	**0.002**	0.747
Apolipoprotein B (mg/dL)	59.5 ± 26.2	80.1 ± 26.9	**0.003**	63.8 ± 27.2	83.4 ± 25.3	**0.015**	0.956
SOFA score	6.5 (5.0)	2.0 (3.0)	**<0.001**	5.0 (5.0)	2.0 (3.0)	**<0.001**	0.271

Values are presented as the mean ± standard deviation (SD) for normally distributed parameters (i.e., albumin, transferrin, Apo AI, and Apo B) and as the median (IQR) for those parameters without a normal distribution. Within–between interactions (treatment × time) were analyzed by a mixed-model ANOVA, and within-group variations were calculated using a *t*-test for paired samples in those parameters with a normal distribution, whereas *p* values for parameters without a normal distribution were calculated using a Wilcoxon signed-rank test. *p* < 0.005 was considered statistically significant. ALT, alanine aminotransferase; AST, aspartate transaminase; GGT, gamma-glutamyltransferase; IQR, interquartile range; OO-ILE, olive-oil-based intravenous lipid emulsion; SMOF-ILE, soybean oil, medium chain triglycerides, olive oil, and fish oil intravenous lipid emulsion; SOFA, sequential organ failure assessment.

**Table 3:** Evolution of clinical characteristics and outcomes of the study population.

	OO-ILE (*n* = 29)	SMOF-ILE (*n* = 25)	*p*
Mechanical ventilation (days)	7.0 (8.5)	1.0 (24.0)	0.185
CVC (days)	16.5 (18.8)	18.0 (16.5)	0.908
ICU length of stay (days)	13.0 (20.2)	17.0 (21.5)	0.964
Hospital length of stay (days)	30.5 (22.8)	24.0 (16.5)	0.748
Days of PN	10.0 (6.0)	10.0 (7.5)	0.693
VAP	2	3	0.222
CAUTI	2	2	1
CRBSI	4	3	0.535
Surgical Infection	3	4	1
Exitus	7	9	0.446

Data are presented as the median (IQR) or frequencies. A normal distribution of quantitative data was analyzed by a Shapiro–Wilk test, and statistical differences were analyzed using a Wilcoxon signed-rank test. The differences between categorical data were analyzed using a Pearson’s Chi-squared test. *p* < 0.005 was considered statistically significant. CAUTI, catheter-associated urinary tract infection; CRBSI, catheter-related blood stream infection; CVC, central venous catheter; IQR, interquartile range; MV, mechanical ventilation; OO-ILE, olive-oil-based intravenous lipid emulsion; SMOF-ILE, soybean oil, medium chain triglycerides, olive oil, and fish oil intravenous lipid emulsion; VAP, ventilator-associated pneumonia.

## Data Availability

Data are available from the authors upon reasonable request.

## Supplementary Materials

The following supporting information can be downloaded at: https://www.mdpi.com/article/10.3390/ijms252111739/s1. Ref. [41] is cited in Supplementary Materials file.
